# A novel modelling approach to quantify the response of dairy goats to a high-concentrate diet

**DOI:** 10.1038/s41598-020-77353-y

**Published:** 2020-11-23

**Authors:** Masoomeh Taghipoor, Maud Delattre, Sylvie Giger-Reverdin

**Affiliations:** 1 Université Paris-Saclay, INRAE, AgroParisTech, UMR Modélisation Systémique Appliquée aux Ruminants, 75005 Paris, France; 2 Université Paris-Saclay, INRAE, MaIAGE, 78350 Jouy-en-Josas, France

**Keywords:** Animal physiology, Computer modelling

## Abstract

High-producing ruminants need high-concentrate diets to satisfy their nutrient requirements and meet performance objectives. However, such diets induce sub-acute ruminal acidosis (SARA), which will adversely affect dry matter intake and lead to lower production performance. This work develops a novel modelling approach to quantify the capacity of dairy goats to adapt to a high-concentrate diet challenge at the individual level. The animal model used was dairy goats (from Saanen or Alpine breed), and rumen pH was used as the indicator of the response. A three-step modelling procedure was developed to quantify daily scores and produce a single global index for animals’ adaptive response to the new diet. The first step summarizes the post-prandial kinetics of rumen acid status using three synthetic variables. In the second step, the effect of time on the response of goats is described, in the short and long terms. In the last step, a metric based on phase trajectories ranks goats for their resilience capacity. This modelling procedure showed a high variability among the goats in response to the new diet, highlighting in particular their daily and general strategies to buffer the effect of the diet change. Two main categories of adaptive strategies were observed: (i) acid status increased, but the goats tried to minimize its variations, and (ii) acid status oscillated between increases and decreases. Such phenotyping, alongside other behavioral, digestive, and metabolic measures, can help to determine biomarkers of goats’ capacity to adapt to diets of higher nutritive value and to increase production performance without compromising their health status. Quantifying the capacity of goats to buffer the effect of highly fermentable diets helps to better adapt feed to animals in precision livestock farming. This procedure is generic and can be adapted to any indicator of animal health and performance. In particular, several indicators can be combined to assess multi-performance, which is of major interest in the context of selection for robust animals.

## Introduction

Although ruminants can valorise fibrous low-quality diets^1^, high-producing ruminants need high-concentrate diets to satisfy their nutrient requirements. Such diets are often rich in highly and rapidly fermentable carbohydrates, and so increase the production of volatile fatty acids, which in turn decreases rumen pH to non-physiological levels^2^. When rumen pH falls below a threshold often set at around 6.0, cellulolysis is inhibited and therefore dry matter intake is depressed^3^. Animals are then considered to be in a condition of sub-acute rumen acidosis (SARA), some physiological and pathological consequences of which have been extensively reviewed by Owens et al*.*^4^ The main effects of SARA are decreased feed efficiency and a modification of various production traits. Daily rumen acid status pattern varies considerably between diets and between animals fed the same diet^5^. It is well known that within a herd, some animals are susceptible to SARA when fed a high-concentrate diet, while others are tolerant^6^. Several indicators have been proposed to evaluate this pattern and the occurrence of SARA: initial and final pH values, amplitude of pH variations, area under a given pH threshold and the pH curve, time under a given threshold, etc.^7^ However, to our knowledge, no quantification of individuals’ response in terms of rumen acid status has yet been carried out. Assigning an index to each individual is a step forward in the search for non-invasive biomarkers of the animal’s capacity to buffer the effect of a high-concentrate diet. The comprehension of mechanisms underlying this capacity will be assisted by associating this global index with other behavioural and physiological measures in response to perturbation^4,6,8^. Ongoing environmental, economic and societal changes have meanwhile led animal scientists and selection companies to consider new strategies to select more robust animals^9–11^. The index described here allows ranking of ruminants for their capacity to buffer the effect of a highly fermentable diet, and subsequently refining strategies to select for animals more resistant to SARA. Finally, in livestock precision farming, this index helps to adapt feed composition to an animal’s rumen capacity and thereby efficiently valorise food composition^12^.

The main objective of this study was to develop a modelling approach to quantify animals’ capacity to regulate rumen acid status in response to a high-concentrate diet challenge, at the individual level. The animal model used was the dairy goat (from Alpine or Saanen breeds). The indicator of rumen acid status was pH, the post-prandial kinetics of which were measured throughout the 9 days of sampling. Various authors have quantified animals’ response to different types of perturbations, such as feed restriction, weaning, response to mycotoxin challenges or general perturbations with unknown origins^13–16^. In these studies, the indicator of the animal response (body weight, feed intake, biochemical samples in plasma, milk yield, etc.) was measured at one given time scale (daily, weekly, etc.). The main difficulty in quantifying goats’ response in terms of pH in the present trial was the presence of two different and imbricated sampling time scales, namely the hourly scale of post-prandial pH and the daily scale of the experimentation. To address this problem, an original three-step modelling procedure was developed. Firstly, the variations of the post-prandial acid status pattern were modelled to obtain some biologically relevant synthetic variables describing the post-prandial kinetics. Secondly, a mixed model was developed for each of the selected synthetic variables to study the short- and long-term effects of the new diet. It was hypothesised that goats would be able to regulate their rumen pH in the short term, whereas in the long term it might become more and more difficult to overcome the challenge of the high-concentrate diet. Finally, the variables with significant long-term effects were used to develop a modelling approach drawing on health trajectories^17^ to quantify individual goats’ capacity to meet the challenge of a new diet. Using this approach, it was possible to assign a single numerical index to each goat describing its performance throughout the period of experimentation.

Since working with the small values of hydrogen ion concentration (${[\text{H}}^{+}],$ moles per litre) was inconvenient, Sørensen^18^ advocated a pH scale, where $\text{pH}= -{\text{log}}_{10}[{\text{H}}^{+}]$. Although this is a useful scale to measure rumen acidity, it may lead to errors in statistical tests and mathematical calculations^19^. In this work, values of ${[\text{H}}^{+}]$ were therefore used for model development and statistical tests. A comparison between results obtained using ${[\text{H}}^{+}]$ and $\text{pH}$ is made in “Results” section.

## Materials and methods

The goats were cared for and handled in accordance with the French legislation on animal experimentation and in line with the European Convention for the Protection of Vertebrates Used for Experimental and Other Scientific Purposes (European Directive 86/609). All experimental procedures followed the guidelines for the care and use of experimental animals and were approved by the local ethics committee (Comité d’Ethique en Expérimentation Animale, COMETHEA 45, registered as 15-04). This study was conducted, from mid-April to mid-May 2015, at the experimental farm of the Inrae-AgroParisTech MoSAR Research Unit (Thiverval-Grignon, France, 48°51′ N 1°55′ E, 70 m a.s.l.).

### Experimental design

Eight rumen-cannulated dairy goats (four Alpine and four Saanen) were habituated to a total mixed ration with a low concentrate level (20% on a dry matter basis). This was abruptly switched to a total mixed ration with a high concentrate level (50%). The two diets were formulated to be iso-protein (iso-PDI or truly digestible protein) according to the renewed French Inrae System for ruminants^20^. Goats were fed in accordance with their nutritional requirements and in average 13% of refusal was observed (13% ± 8%). This means that goats were able to express their natural feeding behaviour. Moreover, the diet was a TMR (Total Mixed Ration) to prevent from sorting.

**Figure 1:**
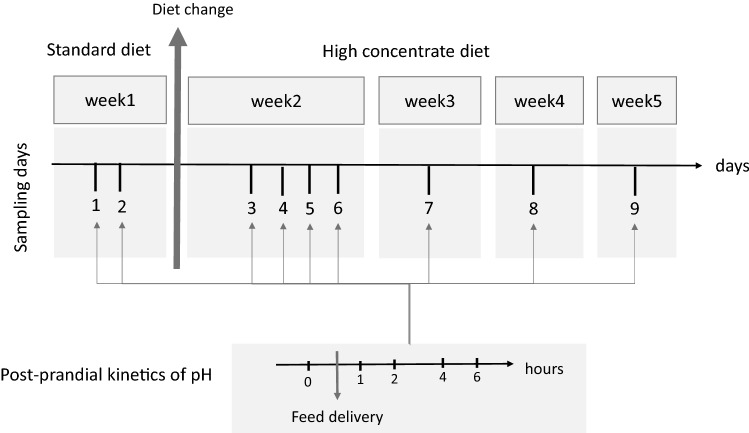
Experimental design. Goats were fed the standard diet in the first week (the first 2 days). Their diet was changed to the high-concentrate diet in the next 4 weeks of experimentation (Days 3 to 9). At each sampling day, pH was recorded before and 1, 2, 4 and 6 h after feed delivery. The presence of two embedded dynamics can be seen in the scheme: one for post-prandial kinetics of pH, the second for the sampling days.

Rumen fluid was sampled through the cannula before and 1, 2 , 4 and 6 h after the morning feed delivery on 2 days before the change of diet, $${d}_{1}$$ and $${d}_{2}$$ (where $${d}_{2}={d}_{1}+3)$$, then on four consecutive days following the diet change, $${d}_{3}, {d}_{4}, {d}_{5}$$ and $${d}_{6}$$, and finally once a week for 3 weeks, $${d}_{7}, {d}_{8}$$ and $${d}_{9}$$, where $${d}_{7}={d}_{6}+4, {d}_{8}={d}_{7}+6,$$ and $${d}_{9}={d}_{8}+7$$. Rumen pH was measured immediately after sampling. Figure 1 illustrates the experimental design.

To allow for the time interval between milking, feed was delivered shortly after milking (7 a.m. and 3 p.m.), with 1/3 of the total ration in the morning and 2/3 in the afternoon. Diet samples were collected for 9 days corresponding to rumen sampling. They were analysed separately for dry matter (DM)^21^ and cell wall components estimated by the neutral detergent fibre method (NDF) of Van Soest and Wine^22^ modified by Giger et al*.*^23^ These last authors suggest using a heat-stable alpha-amylase without sodium sulphite and decalin as proposed by Robertson and Van Soest^24^. Lignocellulose (ADF) and acid detergent lignin were obtained using a sequential approach on the NDF residue^23^. Starch content was analysed according to AFNOR (1997) method^25^. Total N was determined by the Dumas technique^26^, and crude protein was estimated as 6.25 N. The nutritive value of the diets was obtained according to the renewed French INRA System for ruminants^20^. Details are provided in supplementary Table S1.

### Modelling procedure

Values of $\text{pH}$ were first transformed by applying the logarithmic function to obtain the values of hydrogen ion concentration ${[\text{H}}^{+}]$ ($\text{pH}=-{\text{log}}_{10}{[\text{H}}^{+}]$).

The main difficulty in developing a model to describe such data was the presence of two different imbricated time scales, namely several weeks of experimentation versus the post-prandial kinetics scale (hours, Fig. 1). Accordingly, the post-prandial kinetics of ${[\text{H}}^{+}]$ were first summarised with small numbers of synthetic variables, which were then integrated into the weekly data analysis.

The modelling procedure is detailed in three steps. The first step describes the procedure to determine the synthetic variables, the second step explains the development of the mixed model to study the effect of time on goats’ response, and in the third step, an original method is introduced to quantify animals’ capacity to adapt to the new diet.

### Step 1: Synthetic variables to describe the post-prandial kinetics of $[{\mathbf{H}}^{+}]$

To determine the synthetic variables, the post-prandial kinetics of ${[\text{H}}^{+}]$ were described with a quadratic function,

$${[\text{H}}^{+}]\left(t\right)=a{t}^{2}+bt+c, \quad \text{Model }\,(1.1)$$

where *t* is time of sampling and *a*, *b* and *c* are the quadratic function coefficients. This function was re-parametrised to obtain a function with biologically meaningful parameters^27^,

$${[\text{H}}^{+}]\left(t\right)=f\left(t,{v}_{0}, A, R\right), \quad \text{Model }\,(1.2)$$

where ${v}_{0}, A$ and $R$ are the new parameters of the model, ${v}_{0}$ is the initial value of ${[\text{H}}^{+}]$, $A$ is the amplitude of acidosis or the extent of the deviation from ${v}_{0}$, and $R$ represents the recovery capacity of the animal 6 h after feed delivery (see supplementary Eq. 1 for the re-parametrisation).

**Figure 2:**
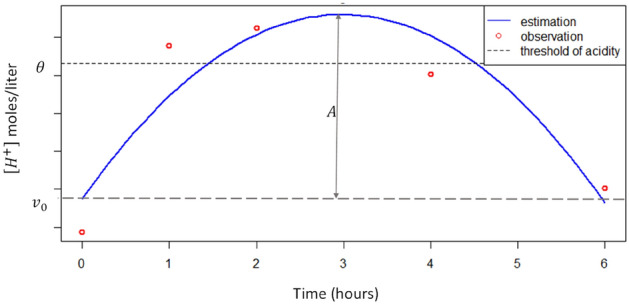
Illustration of the parameters of the quadratic function used to fit post-prandial ${[\text{H}}^{+}]$ curves. Parameter ${v}_{0}$ is the initial value of ${[\text{H}}^{+}]$, *A* is the maximum deviation from the initial value and represents the difference between ${\text{max}[\text{H}}^{+}]$ and ${v}_{0}$. Parameter *R* is not illustrated in the figure and is the percentage of recovery after 6 h. $\theta$ is the threshold of acidity.

The threshold of acidosis $\theta$ is the value of ${[\text{H}}^{+}]$ above which the animal is assumed to be in a condition of rumen acidosis (Fig. 2). This model was then fitted to all the individual post-prandial kinetics of ${[\text{H}}^{+}]$ to estimate the triplet ${v}_{0},A,R$. Besides obtaining biologically meaningful parameters, the re-parametrisation of the quadratic function helped to avoid the statistical difficulties arising from the initiation of the model parameters $(a,b,c)$ for 9 days of post-prandial kinetics of eight goats.

Besides ${v}_{0},A,R$, other synthetic variables can be calculated from Model 1.2. The variable duration ($\text{dur}$) stands for the duration of acidosis when ${[\text{H}}^{+}]$ exceeds $\theta$, amplitude of acidosis ($\text{AmpAc}$) is associated with the difference between the maximum of ${[\text{H}}^{+}]$ and $\theta$, and the variable ${\text{var}}_{\text{last}}$ is the estimation of ${[\text{H}}^{+}]$ 6 h after the feed delivery. Variables $\text{AmpAc}$ and $\text{dur}$ are dependent on the values of $\theta$.

### Step 2: Statistical analysis on synthetic variables

To study the effect of time on the goats’ response to the dietary change during the 5 weeks of experimentation, a mixed model was developed for each of the synthetic variables (Model 2), with individual goats as random effect. Fixed effects were the qualitative variable “week” for the weeks of sampling, associated with the response of the animal in the long term. The numerical variable “days”, which represented days of sampling, was introduced to describe the short-term response of goats during the first week. The variable “days” was taken to be positive after the delivery of high-concentrate diet (Day 1 was the first day of delivery of the high-concentrate diet). The random effect “goats” helped to quantify the contribution of individual variability among the goats to the total variance of the model^28^.

$${v}_{ijk}={\alpha }_{j}+{G}_{i}+\beta .\left({\text{days}}_{k}.{1}_{j=2}\right)+{\epsilon }_{ijk} \quad (\text{Model }2)$$

$${G}_{i}\in \mathcal{N}\left(0,{\sigma }_{A}^{2}\right), \quad i=1,\dots ,8$$

$${\epsilon }_{ijk}\in \mathcal{N}\left(0,{\sigma }^{2}\right), \quad j=1, \dots , 5, \quad k=1,\dots ,9.$$

where $i$ stands for the number of animals, $j$ the number of weeks, and $k$ the number of days of sampling, ${v}_{ijk}$ is the synthetic variable, ${G}_{i}$ is the random intercept for goat $i$, ${\alpha }_{j}$ is the fixed effect of $"\text{week}"$ and $\beta$ is the fixed effect of $"\text{days}"$. The indicator function $({1}_{j=2}$) indicates that the effect of $\beta$ is applied only during the second week of sampling (first week after the delivery of high-concentrate diet),

$${1}_{j=2}=\left\{\begin{array}{c}1, j=2 \, \\ 0, j\ne 2\end{array}\right.$$

**Figure 3:**
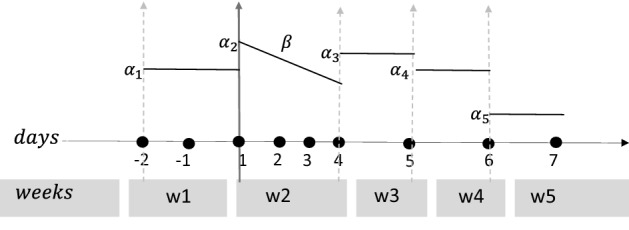
Illustration of Model 2. “Week” is a factor and varies from w1 to w5, “days” is a numerical variable associated with the days of sampling. As seen in the graph, ${\alpha }_{j}$ determines the intercept associated with each week and $\beta$ is the slope of daily variation of the synthetic variable during the first week after the diet change. Slope $\beta$ is applied only during week 2 because of the use of the indicator function.

The model has a random intercept for the difference among goats at the beginning of experimentation. Figure 3 illustrates the fixed effect of the model.

### Step 3: Quantification of goats’ response

**Figure 4:**
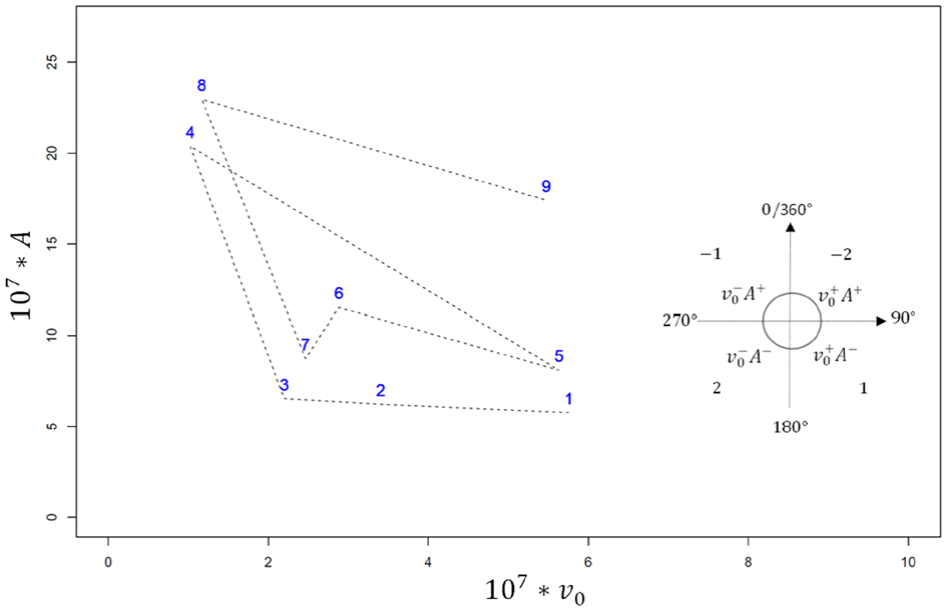
Example of a phase plane of ${v}_{0}$ and $A$, representing variations of *A* as a function of ${v}_{0}$, rescaled by ${10}^{7}$ for clarity. Numbers (1 to 9) on the curve are days of sampling. A score ${s}_{i\to i+1}$ is assigned to the variation from day ${d}_{i}$ to day ${d}_{i+1}$, for $i\in \{1, \dots ,8\}$. The small plane on the right-hand side of the graph illustrates the assignment of scores. The score of − 2 is assigned to ${v}_{0}^{+}{A}^{+}$ describing an increase in both variables, a score of − 1 is assigned to ${v}_{0}^{-}{A}^{+}$, i.e. decreasing ${v}_{0}$ and increasing $A$, a score of 1 is associated with ${v}_{0}^{+}{A}^{-}$ describing increasing ${v}_{0}$ and decreasing $A$, and finally 2 for the decrease in both variables. Each score is then weighted by ${\omega }_{i\to i+1}$ the relative Euclidian distance between days ${d}_{i}$ and ${d}_{i+1}$, calculated as the Euclidian distance between the 2 days divided by (${d}_{i+1}-$ ${d}_{i}$). For this curve the set of scores can be written {− 1 ${\omega }_{1\to 2}$,− 1 ${\omega }_{2\to 3}$,− 1 ${\omega }_{3\to 4}$,1 ${\omega }_{4\to 5}$,− 1 ${\omega }_{5\to 6}$,2 ${\omega }_{6\to 7}$,− 1 ${\omega }_{7\to 8}$,1 ${\omega }_{8\to 9}$}. The adaptive capacity index associated with this animal can then be defined as the sum of the scores of the 9 days of sampling.

To quantify individual capacity to adapt to the new diet, a method based on health trajectories^17,29^ was developed and applied on synthetic variables with a significant long-term effect. In this method, for a given animal, the phase space for synthetic variables was plotted. Let us consider the example of the two-dimensional phase space (phase plane) for variables ${v}_{0}$ and $A$ (Fig. 4). Based on daily variations of both synthetic variables under study for $i\in \{1, ..,8\}$, a daily score ${s}_{i\to i+1}$ from − 2 to 2 is associated with an animal’s response from day ${d}_{i}$ to ${d}_{i+1}$. For both variables, a decrease is associated with an expected adaptive behaviour, and conversely, an increase is associated with a non-capacity to adapt (see Table 1 for the definition of each synthetic variable). Therefore, if both variables decrease, the score is 2, and if both variables increase, the score is − 2. An increase in ${v}_{0}$ and a decrease in $A$ is associated with the effort of the animal to adapt, i.e., despite the increase in the initial value ${v}_{0}$, the maximum has not changed or is decreased, which results in a decrease in $A$: therefore, to record the effort of adaptation, the score is 1. Finally, the score of − 1 is assigned if despite the decrease in ${v}_{0}$, the value of $A$ increases (maximum post-prandial ${H}^{+}$ increases). To consider the variations of synthetic variables and the number of days between two sampling days ${d}_{i}$ and ${d}_{i+1}$, the relative Euclidian distance in the phase plane was calculated and used as a weighting associated with daily scores (Model 3).

**Table 1:** Two groups of candidate synthetic variables to characterise the post-prandial kinetics of ${[\text{H}}^{+}]$.

Group 1. parameters of the model		
Synthetic variables	Initial values	Definition
${v}_{0}$ (moles/litre)	${[\text{H}}^{+}]\left({t}_{0}\right)$	Initial value of ${[\text{H}}^{+}]$ as estimated by the model
$A$ (moles/litre)	$\text{max}\left({[\text{H}}^{+}]\right)-{v}_{0}$	Difference between maximum of ${[\text{H}}_{\text{estim}}^{+}$ ]and ${v}_{0}$
$R$ (dimensionless)	$\frac{\text{max}\left({[\text{H}}^{+}]\right)-{v}_{0}}{\text{max}\left({[\text{H}}^{+}]\right)-{v}_{\text{last}}} \times 100$	The capacity of a goat to reach its initial ${[\text{H}}_{\text{estim}}^{+}]$ at t = 6 h

Group 2. calculated from the quadratic function for $\theta =5.5$		
Synthetic variables	Formula	Definition
$\text{dur}$ (hours)	$\text{Time }([{\text{H}}_{\text{estim}}^{+}]<\theta )$	Time interval of acidosis measured by the time that ${[\text{H}}_{\text{estim}}^{+}]$ is under the threshold $\theta$ of acidity
$\text{AmpAc}$ (moles/litre)	$\left({\text{max}([\text{H}}_{\text{estim}}^{+}]\right)-\theta )$	Amplitude of acidity, the difference between $\theta$ and ${\text{max}([\text{H}}_{\text{estim}}^{+}]$
${v}_{\text{last}}$	${[\text{H}}^{+}]\left({t}_{\text{last}}\right)$	The value of ${[\text{H}}^{+}]$ at *t* = 6 h as estimated by the model

$[{\text{H}}_{\text{estim}}^{+}]$ is the estimation of ${[\text{H}}^{+}]$ by the model. Variable $\text{dur}$ is the time interval of acidosis where $[{\text{H}}_{\text{estim}}^{+}]<\theta$, and $\text{AmpAc}$ is the extent of the deviation from the threshold $\theta =5.5$. Variable ${v}_{\text{last}}$ is the value of ${\text{H}}^{+}$ at *t* = 6 h, as estimated by the model. In contrast to Group 2, the synthetic variables of Group 1 are independent of the value of $\theta$.

$${\omega }_{i\to i+1}=\frac{1}{{d}_{i+1 }-{d}_{i}}*\sqrt{{\left(A\left({d}_{i+1}\right)-A\left({d}_{i}\right)\right)}^{2}+{\left({v}_{0}\left({d}_{i+1}\right)-{v}_{0}\left({d}_{i}\right)\right)}^{2}} \quad (\text{Model }3)$$

Using this method, a daily index and a global index of animal adaptive capacity are defined: daily index is the daily score associated with each animal, and global index is calculated as the sum of daily scores from day ${d}_{2}$ to ${d}_{9}$ (first day of diet change is ${d}_{3}$),

$${d}_{i\to i+1}= {s}_{i\to i+1 }*{\omega }_{i\to i+1}$$

$$\text{GI}= {\Sigma }_{2}^{8}{d}_{i\to i+1},$$

where, ${d}_{i\to i+1}$ stands for the daily score assigned to the daily response of a goat, and GI is the global index for each goat.

### Statistical analysis

Software R version 3.5.3 (R core team 2019; https://www.R-project.org/)^30^ was used for all statistical analyses. For correlation analysis, because the distribution of synthetic variables was not Gaussian, the Spearman correlation test was used. To study the effect of fixed variables of the model, the likelihood ratio test (ANOVA function in R) was used. When significant effects of weeks were found, a post hoc comparison test with FDR correction was applied (emeans package^31^). The function nls of package stat was used to estimate the parameters of the quadratic function (Model 1.2). For each of the synthetic variables, the Gaussian distribution was checked with a Shapiro–Wilk test^32^. In the absence of normality, a box-cox transformation was applied to determine the adequate power transformation, using the boxcox function of R. Finally, the function lme4 was used for the mixed models^33^.

## Results

**Figure 5:**
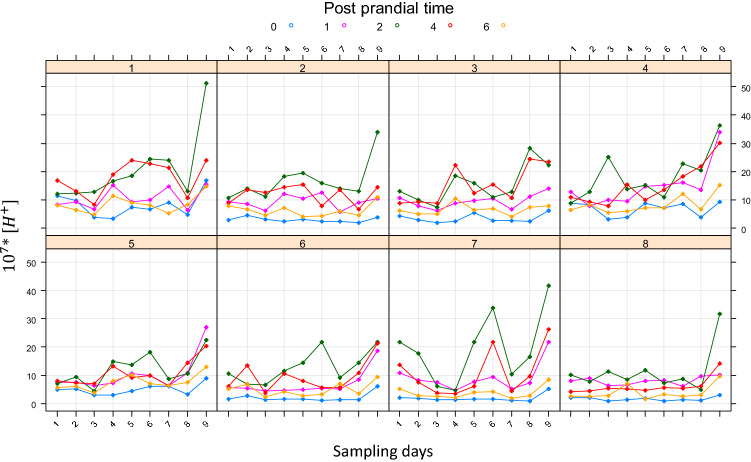
Individuals’ daily variations of ${[\text{H}}^{+}]$. Each panel represents the post-prandial kinetics of individual goats along the 9 days of sampling. Different colours are associated with different time points of post-prandial kinetics of ${[\text{H}}^{+}]$. For clarity, values of ${[\text{H}}^{+}]$ are rescaled by ${10}^{7}$.

Figure 5 shows the postprandial kinetics of ${[\text{H}}^{+}]$ along the 9 sampling days of the trial, for all the goats. No apparent health problem was observed, even though the range of rumen pH observed varied from 5.29 to 7.07 (see Supplementary Fig. S1 online).

### Step 1: Synthetic variables

**Figure 6:**
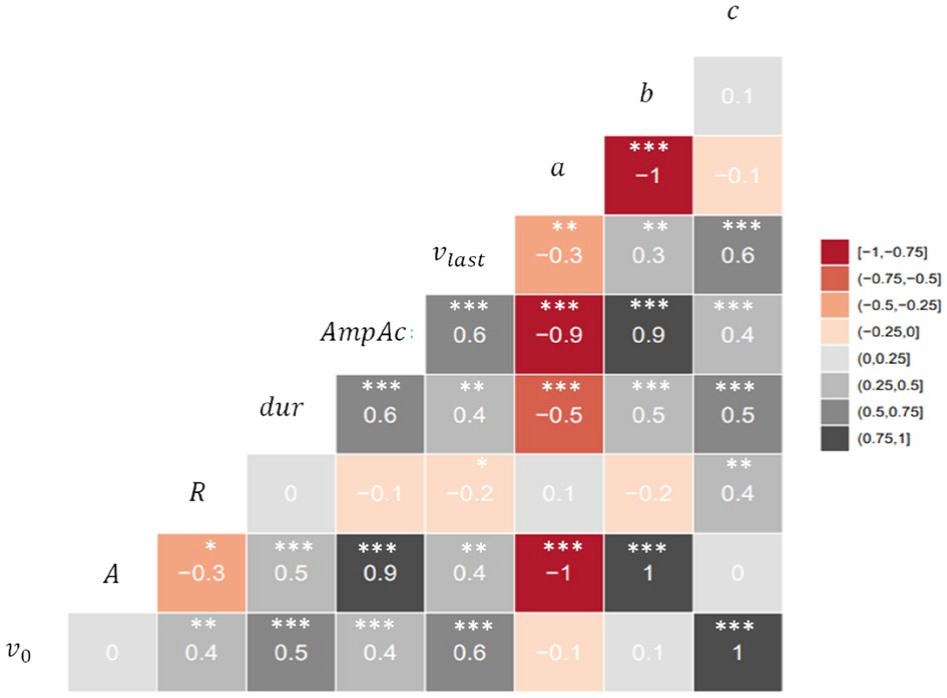
Graph of correlation among synthetic variables. Correlation values and their significance are noted. Symbols *, ** and *** mean *p* values smaller than 0.05, 0.01, and 0.001, respectively. Because the distribution of synthetic variables was not Gaussian, the Spearman correlation test was used. $a$, $b$ and $c$ are the coefficients of the original quadratic function. $A$, ${v}_{0}$ and $R$ are parameters of the new re-parametrised function. $\text{AmpAc}$ and $\text{dur}$ are parameters calculated after fitting the model to individual ${[\text{H}}^{+}]$ daily kinetics.

Figure 6 shows the correlation among different synthetic variables introduced in Table 1 (see supplementary Table S1 online for descriptive statistics of the synthetic variables). There was a high positive correlation between $A$ and $\text{AmpAc};$ both indicators of the maximum post-prandial ${[\text{H}}^{+}]$ deviation (*r* = 0.89, *n* = 72). The variable $a$ represents the curvature (second derivative) of the quadratic function, and was highly correlated with $\text{AmpAc}$ and $A$, explaining the fact that the larger $a$, the greater the deviation of the function from the origin (with respectively, *r* =  − 0.90 and *r* =  − 0.96, *n* = 72). Different thresholds of acidity $\theta$ are reported in the literature^34^, and the evolution of the synthetic variables should be considered in relation to $\theta$. For example, $\text{AmpAc}$ and $\text{dur}$ are dependent on the values of $\theta$. Increasing $\theta$ will down-shift both $\text{AmpAc}$ and $\text{dur}$. This was expected, given the definitions of these variables in Table 1. Instead of using $\theta$-dependent variables, three synthetic variables ${v}_{0}$, $A$ and $R$ were used to describe the post-prandial ${[\text{H}}^{+}]$ kinetics for each goat.

### Step 2: Statistical analysis on synthetic variables

The Shapiro test showed that none of the three variables ${v}_{0},\;A \;\text{and} \;R$ were Gaussian $\left(p<0.05\right).$ A power transformation was applied (box-cox transformation), and then the mixed model (Model 2) was used on power transformed variables ${v}_{0}^{{\lambda }_{1}},\;{A}^{{\lambda }_{2}} \;\text{and} \;{R}^{{\lambda }_{3}}$ . When significant differences were detected, differences among means were tested using Tukey’s comparison test with FDR correction (false discovery rate, emmeans package of R). For all variables, the Gaussian distribution of residuals and the heteroscedasticity of the model were checked. Results are presented in Table 2.

**Table 2:** Effect of the shift from the standard to a high-concentrate diet on synthetic variables describing the post-prandial kinetics of rumen hydrogen ion concentration $[{\text{H}}^{+}]$ along the 5 weeks of experimentation.

	Weeks					p-values	
	W1	W2	W3	W4	W5	Week	Days
${v}_{0}^{{\lambda }_{1}}$	0.052^a^	0.046^c^	0.049^ac^	0.047^c^	0.054^b^	< 0.001	0.11
${A}^{{\lambda }_{2}}$	0.016^a^	0.017^ab^	0.018^a^	0.020^bc^	0.023^c^	< 0.001	0.11
${R}^{{\lambda }_{3}}$	4.40	3.98	4.03	4.08	4.29	0.36	0.37

Means without a common superscript letter differ significantly (*p* < 0.05, a-d within a row). ${A}^{{\lambda }_{1}}, {v}_{0}^{{\lambda }_{2}}$ and ${R}^{{\lambda }_{3}}$ are the power transformed values of *A* and ${v}_{0}$ and $R$. W1 is associated with the time interval of distribution of the standard diet, and W2 to W5 with the distribution of the high-concentrate diet. Week and days describe the significance of fixed effects of the mixed model.

Variable ${v}_{0}^{{\lambda }_{1}}$ for ${\lambda }_{1}=0.20$ was not affected by the fixed effect of “days” (${\chi }^{2}\left(1\right)=38.7, p=0.11$), but was significantly affected by the fixed effect of “week” (${\chi }^{2}\left(1\right)=2.51, p<0.001)$. The synthetic variable ${A}^{{\lambda }_{2}}$ for ${\lambda }_{2}=0.28$ was not affected by the effect of “days” (${\chi }^{2}\left(1\right)=2.52 , p=0.11)$, but was significantly affected by the fixed effect of “week” (${\chi }^{2}\left(1\right)=28.4, p<0.001)$. The anova test showed that the effects of “days” and “week” were not significant (*p* > 0.05) on post-prandial recovery capacity $R$.

The contribution of the individual variability to the total variance of each of the synthetic variables can be presented as the coefficient $\rho =\frac{{\sigma }_{A}^{2}}{{\sigma }^{2}+{\sigma }_{A}^{2}}$^28^. ${\sigma }_{A}^{2}$ is the variance associated with the goat effect (between-goats variation) and ${\sigma }^{2}$ is the variance of residuals (within-goats variation). For the synthetic variable $A$, 17% of the variation was due to between-goats variability $(\rho = 0.17)$, more than 50% of the total variance of ${v}_{0}$ was described by the goat effect $\left(\rho = 0.56\right),$ and only 10% of total variance of $R$ was due to individual variability.

### Step 3: Quantification of goats’ response

Only synthetic variables with a significant long-term effect ($${v}_{0}$$ and $$A)$$ were used to calculate goats’ capacity to adapt to the diet change. Table 3 gives daily scores and global index for each goat. For a better presentation of the results, all scores were rescaled by $${10}^{7}$$: the higher the global index, the better the adaptive capacity of the animal. The global index for all the goats was negative. The results in Table 3 show six goats with larger global indices (> − 10), while two others had rather small indices (< − 20). Goat 8 had the best adaptive response (least negative), and Goat 1 the poorest response in terms of capacity to adapt to the new diet.

**Table 3:** Daily scores and global index for goats’ response to high-concentrate diet challenge.

Goat #	${d}_{1\to 2}$	${d}_{2\to 3}$	${d}_{3\to 4}$	${d}_{4\to 5}$	${d}_{5\to 6}$	${d}_{6\to 7}$	${d}_{7\to 8}$	${d}_{8\to 9}$	Index
1	0.89	− 5.64	− 17.74	− 2.22	− 0.64	1.1	2.9	− 4.19	− 26.42
2	− 1.09	− 2.14	− 6.36	− 1.34	6.2	− 1.15	0.82	− 2.69	− 6.66
3	− 0.8	− 1.24	− 13.91	13.12	− 4.39	1.41	− 2.38	0.88	− 6.51
4	− 0.72	− 9.5	− 1.3	10.84	4.06	− 3.87	− 1.53	− 2.71	− 4.01
5	− 0.27	− 2.38	− 6.83	5.64	− 3.77	2.98	− 1.53	1.19	− 4.7
6	− 1.25	5.99	− 5.78	0.6	− 3.97	1.54	− 0.99	− 1.7	− 4.31
7	3.08	7.8	− 1.11	− 16.19	− 14.88	4.89	− 0.96	− 4.08	− 24.53
8	0.7	− 4.6	2.57	− 3.76	5.77	− 0.54	0.46	− 3.67	− 3.76

${d}_{i\to i+1}$ is the value assigned to the quality of the response of each goat from day ${d}_{i}$ to ${d}_{i+1}$ for $i\in \{1, ..,8\}.$ Decreasing values of $A$ from day ${d}_{i}$ to ${d}_{i+1}$ are associated with the capacity to adapt to the new diet, which is recognised with a positive daily score. Conversely, increasing values of $A$ from day ${d}_{i}$ to ${d}_{i+1}$ show that goats had difficulties adapting to the new diet, and negative daily scores are assigned. The value of the score depends on the variation of ${v}_{0}$ from day ${d}_{i}$ to ${d}_{i+1}$ and also the weighting ${\omega }_{j}$ associated with the relative Euclidian distance between ${d}_{i}$ and ${d}_{i+1}$.

**Figure 7:**
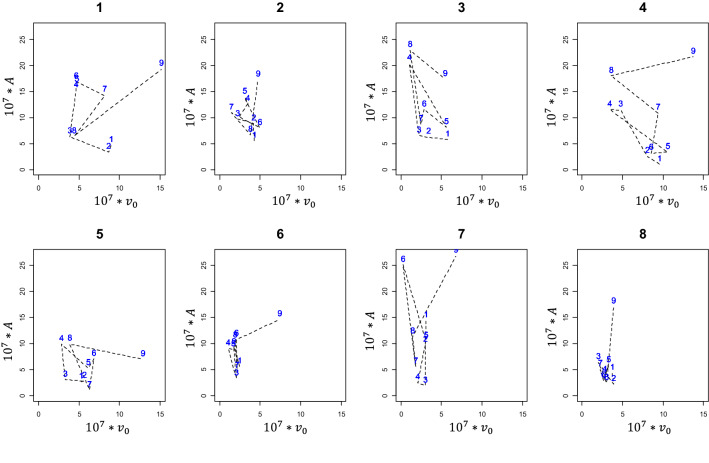
Phase trajectories of goats in response to the high-concentrate diet challenge. Each graph shows the trajectories of each individual goat in terms of $A$ and ${v}_{0}$, represented by dashed lines. Goat numbers from 1 to 8 are stated above each graph. Sampling days (from 1 to 9) are in blue inside each graph. Both variables ${v}_{0}$ and $A$ are rescaled by ${10}^{7}$.

Figure 7 shows two main strategies of response in goats. The first strategy involved keeping variations of ${[\text{H}}^{+}]$ as small as possible, resulting in smaller Euclidian distances (Goats 8, 2 and 6). The second strategy consisted in an oscillating response, mainly in variations of $A$ (Goats 3 and 5).

### Analysis of pH data

The effect of the diet change was studied on the synthetic variables calculated from pH, for the purpose of comparing with ${[\text{H}}^{+}]$ results.

The initial pH (variable ${v}_{0}$) was significantly affected by the fixed effects of both “week” (${\chi }^{2}\left(1\right)=72.70, p<0.001$) and “days” (${\chi }^{2}\left(1\right)=11.7, p<0.001$). In all, 81% of the variation of ${v}_{0}$ was related to the individual variability. The deviation from the initial pH (variable $A$) was significantly affected by the fixed effect of “week” (${\chi }^{2}\left(1\right)=17.1 , p<0.01$), while the effect of “days” was not significant. Results showed that 38% of the variation of A was related to the individual variability of goats. No effect of diet on the recovery capacity was observed, and only 9% of the total variation was related to individual variability (see supplementary Table S2 online).

## Discussion

An original modelling procedure was developed to quantify goats’ capacity to adapt to the challenge of a high-concentrate diet, at the individual level. Using the metric defined in Model 3, a global index was assigned to each goat, which allowed ranking the goats for their capacity to buffer the effect of the new diet. A daily score was also assigned, which made it possible to compare the evolution of goats’ strategy of response. Results in Table 3 show that Goat 8 had the best adaptive capacity. Although several negative daily scores for this goat could be observed, small variations in ${[\text{H}}^{+}]$ (small Euclidian distance) resulted in a high global index. Despite the close scores of Goats 1 and 7 (the poorest responses), a large difference between their daily scores was found. This indicates that these goats exhibited different underlying mechanisms and daily strategies to adapt to the effect of the high-concentrate diet. The use of daily scores in Table 3, together with other systemic measures (e.g. plasma metabolites, feed intake, rumination, etc.) will help shed light on the underlying mechanisms of animal adaptive strategies.

Differences in daily strategies of response (daily scores) justifies the large between-goats variability of ${v}_{0}$ and A, that has been presented in Result section. The between-goats variability of ${v}_{0}$ is linked to the animal’s rumen microbial community, which ferments the diet, to the buffering effect of saliva, and to the absorptive capacity of the animal’s rumen wall^35^. A large between-goats variability for variable $A$, can be explained by the large variation in rumen acid status for some goats, while it was more stable for others. The rumen acid status has an impact on the rumen microbial community, as a marked drop in pH (a large increase in *A),* which decreases its richness and its diversity, as observed by Zhang et al*.*^36^ in goats fed high-grain diets. It has been shown that for ruminants fed the same diet, some develop acidosis while others keep their neutral $\text{pH}$ as observed in cows^37^ or goats^38^. It seems that feeding behaviour^39^ and rumen adaptive capacity^40^ are among the main factors regulating animals’ response to a change of diet. This link needs to be studied in greater depth, and in particular the link between feeding behaviour and evolution of microbiota, and the link between feeding behaviour and feed efficiency^41^.

The global index was developed for two indicators of the animals’ adaptive capacity. However, this method can be transposed to more than two indicators. In the case of $n$ indicators of performance, an $n$-dimensional phase space is needed instead of a phase plane (in two dimensions). The definition of the metric in Model 3 should thus be slightly modified. In addition, the metric used in this work was weighted by the number of days between two sampling days ${d}_{i}$ and ${d}_{i+1}$, which was a strong assumption made to cancel the effect of variable intervals between different recording days (Fig. 1). The optimal use of this approach would be when the indicator of performance is recorded with the same time intervals. Comparing results of weighted and unweighted metrics (see supplementary Table S3 online), the least adaptive animals kept their rank, but the other animals with better capacity to adapt changed rank. This suggests to be cautious in the use of weighted or unweighted metrics and for adapting them to the traits under study.

The method proposed here draws on the method of health trajectories first introduced by Schneider^29^, and later applied by other authors to distinguish between resistance and tolerance capacities of animals in response to pathogens^17,42^. This is of major interest in refining selection strategies for an improved adaptive capacity in the face of pathogens. In this approach, the phase plane of pathogen load versus an indicator of performance such as body weight, feed intake or other health indicators was plotted. A score was then assigned to each time step depending on the health status of the animal. Using this method, the authors concluded on the dynamic interrelationship between resistance and tolerance in the animal’s response. In our study, the phase plane was presented for two indicators of a goat’s performance, i.e., the phase plane represented a response-response plot, which differed slightly from the strategy of presenting a cause-response plot (e.g. pathogen load versus performance).

Other models have been developed to quantify animals’ response to perturbations, using time series data^13–16^. In all these models, data were available at a high frequency, and on a given time scale, whereas in the present work, the model had to consider two embedded time scales. One originality of this work was the development of a modelling procedure in three steps that enabled these two time scales to be combined to produce one global index. A mathematical model was developed to transform the post-prandial kinetics of ${[\text{H}}^{+}]$ into three synthetic variables. Other authors have also suggested using synthetic variables to describe and summarise the post-prandial kinetics of pH. Molina-Alcaide et al*.*^43^ used the average pH to study the influence of diet on ruminal fermentation. Despite the utility of average pH, it does not consider the dynamic characteristics of post-prandial pH. Other synthetic variables were proposed by Dragomir et al*.*^7^ to analyse the post-prandial pH evolution during the 8 h after delivery of the diet. In their study, two main types of synthetic variables were proposed, the first calculated directly from the initial data and the second corresponding to the parameters of a cubic model (${\text{pH}}_{t}=a{t}^{3}+b{t}^{2}+ct+d)$, or derived therefrom. They finally used a PCA analysis to study the relevant descriptors of the post-prandial evolution of pH. In our approach, given the five post-prandial measures of pH, a quadratic function with three parameters was used to avoid over-parametrisation of the model^44^. The re-parametrisation of the model allowed coefficients *a*, *b* and *c* to be replaced by a set of variables with biological meaningful parameters. The synthetic variable ${v}_{0}$ corresponds to the rumen status of the animal just before the morning feed allowance. Since previous feed delivery was performed 16 h before the morning sampling, ${v}_{0}$ was associated with the basal level, where there is a balance between the production of volatile fatty acids from fermented feeds in the rumen, elimination by absorption through the rumen wall, and travel to the lower digestive tract^45^. The synthetic variable $A$ represents the maximum within-day variation of the acid status of the animal. Large variation of $A$ is therefore associated with large variations in the rumen microflora in its environment, which makes it less efficient, entailing lower feed efficiency^5^. A large variation of A might damage the rumen wall, with serious pathological consequences^46^.

The results confirmed the hypothesis that variables $A$ and ${v}_{0}$ were not affected by the short effect of dietary change (fix effect of “days”), illustrating the capacity of goats to overcome the high-concentrate diet challenge in the short term. These variables were little affected by the fixed effect of “week” before Week 4. This suggests that during the first weeks after the diet change, goats succeeded in regulating ${[\text{H}}^{+}]$. These results can be explained by the ability of goats to modify their feeding behaviour when fed a high-concentrate diet. For example, they could decrease their feed intake, their intake rate and increase the time spent ruminating per unit of intake^38,47^. In some cases, they can also look for fibres, which are less fermentable than starch^39^. Other authors reported some changes in behaviour of cows in cases of disease^48^. At Week 5, a significant increase in $A$ and ${v}_{0}$ was observed. However, the authors suggest that the response of only 1 week is not enough to conclude on adaptive capacity in the long term. For a better understanding of the mechanisms underlying these results, they must be analysed together with feed intake and time spent ruminating. This analysis lay outside the scope of this work, which focused more narrowly on the modelling aspects of merging the time scales and developing the approach to quantifying the global index.

The reported threshold of acidosis $\theta$ ranges from 5.5 to 6.0^49,50^. This should be considered in the definition of synthetic variables. In the model developed here, all three synthetic variables for ${[\text{H}}^{+}]$ are threshold-independent. This means that the analysis is valid whatever the value of the threshold for acidosis. This is of major interest when comparing small and large ruminants. The literature shows that goats have a higher pH (+ 0.4 pH) than cattle when fed diets with similar NDF (neutral detergent fibre) or concentrate percentage^34^, which implies that the threshold under which animals develop acidosis might be different for large and small ruminants.

Importantly, the greater the number of animals being studied, the more meaningful is the between-goats variability and the statistical significance of the results, whence the utility of pooling several trials and performing a meta-analysis on different data sets^51^. Given the small number of goats studied, the statistical power of the tests was estimated. Assuming that the true parameter values were those estimated from the sample, the statistical power for the test of the day effect was around 40% with eight goats. With 20 goats, it reached 70%, while for the week effect, the power was almost 100% even with eight goats. This means a sufficient power for the week effect, but a rather low power for the days effect. This was expected, because of the smaller number of data to estimate the days effect (four observations per goat) than to estimate the week effect.

The differences between results for ${[\text{H}}^{+}]$ and $\text{pH}$ emphasise that the systematic use of pH as an indicator of rumen acidosis can lead to errors, in particular when using statistical tests or mathematical calculations, as explained also by Murphy^19^.

In conclusion, this work proposes a novel modelling approach to quantify the adaptive response of goats to a high-concentrate diet in terms of ${[\text{H}}^{+}]$ variations, sampling at two different time scales. This model assigns daily scores and a single global index to each goat’s response. The method is generic and can be adapted to several indicators of performance, and to other types of perturbations with several sampling time scales.

## Supplementary information

Supplementary Information.

## Data Availability

The R script of the model and data are available in public repository Zenodo at https://doi.org/10.5281/zenodo.3741774.
