# Supplementary material for: A novel modelling approach to quantify the response of dairy goats to a high-concentrate diet
Source: Sci Rep. 2020 Nov 23;10:20376. doi: 10.1038/s41598-020-77353-y (PMC7683544; doi:10.1038/s41598-020-77353-y)
Supplement: Supplementary file 1 — Supplementary Information. [file 41598_2020_77353_MOESM1_ESM.pdf]

Supplementary information

A novel modelling approach to quantify the response of dairy goats to a high-concentrate diet

Taghipoor Masoomeh[1], Delattre Maud[2], Giger-Reverdin Sylvie[1]
[1]Université Paris-Saclay, INRAE, AgroParisTech, UMR Modélisation Systémique Appliquée aux Ruminants, 75005, Paris, France
[2] Université Paris-Saclay, INRAE, MaIAGE, 78350, Jouy-en-Josas, France

Table S1. Descriptive statistics of synthetic variables associated to $[H^+]$. For a better representation, values of variables $v_0, A, AmpAC, v_{last}, a, b$ and c are rescaled (x10^7).

	"Mean"	"sd"	"Min"	"Max"
v_0(moles/liter)	4.507	3.038	0.267	15.187
A(moles/liter)	9.31	6.071	1.04	26.777
R (%)	97.738	49.458	12.14	379.029
dur (hour)	0.111	0.539	0	3
$AmpAc$(moles/liter)	-16.529	7.428	-26.801	6.417
v_{last}(moles/liter)	5.578	3.046	0.443	13.189
a	-1.111	0.696	-3.371	-0.14
b	6.842	4.28	0.826	20.13
c	4.507	3.038	0.267	15.187

Table S2. Effect of the shift from a standard to high concentrate diet on synthetic variables describing the post-prandial kinetics of rumen pH, during the five weeks of experimentation.

	Weeks					p-values	
	W1	W2	W3	W4	W5	week	days
v_0	6.30^{a}	6.59^{b}	6.42^{ac}	6.53^{bc}	6.07^{d}	<0.001	<0.001
A	0.36^{a}	0.57^{b}	0.51^{ab}	0.68^{b}	0.52^{ab}	0.001	0.8
R^{γ_1}	3.02	2.73	2.80	2.70	2.90	0.17	0.43

a–d Within a row, means without a common superscript letter differ (P < 0.05). R^{γ_1} is the power transformed value of R and $\gamma_1 = 0.24$.

Table S3. Daily scores and global index for not-weighted metric

"Goat"	$d_{1\to2}$	$d_{2\to3}$	$d_{3\to4}$	$d_{4\to5}$	$d_{5\to6}$	$d_{6\to7}$	$d_{7\to8}$	$d_{8\to9}$	Index
1	2.68	-5.64	-17.74	-2.22	-0.64	4.39	17.43	-33.56	-37.97
2	-3.28	-2.14	-6.36	-1.34	6.2	-4.59	4.89	-21.48	-24.82
3	-2.4	-1.24	-13.91	13.12	-4.39	5.66	-14.28	7.02	-8.03
4	-2.16	-9.5	-1.3	10.84	4.06	-15.5	-9.21	-21.67	-42.27
5	-0.82	-2.38	-6.83	5.64	-3.77	11.92	-9.16	9.51	4.93
6	-3.74	5.99	-5.78	0.6	-3.97	6.18	-5.96	-13.61	-16.56
7	9.23	7.8	-1.11	-16.19	-14.88	19.57	-5.75	-32.66	-43.22
8	2.11	-4.6	2.57	-3.76	5.77	-2.17	2.77	-29.33	-28.74

Results showed that animals 1 and 7 kept their places as worst responses to the high concentrate diet. However, using this new metric the ranking of the best animals has changed, animals with a decrease in the last step are considered as the best .

Table S4. Ingredient composition, chemical composition, and nutritive values of the diets given successively to eight goats. No additive or ruminal modifiers were added.

Item	Low-Concentrate Diet	High- Concentrate Diet
**Ingredients (% DM)**		
Meadow hay	24	35
Chopped dried alfalfa	28	0
Pressed sugar beet pulp	28	15
Compound feed	20	50
**Chemical composition (% DM)**		
CP	13.0	12.9
NDF	43.2	40.4
ADF	23.9	21.2
ADL	3.7	3.8
Starch	10.4	18.5
**Nutritive** value		
UFL (/kg DM)	0.83	0.88
PDI (/kg DM)	95.2	94.7

CP (crude protein) estimated as 6.25 N measured by the Dumas technique[1]. NDF (Neutral Detergent Fibre) of Van Soest and Wine (1967)[2]. ADF (Acid Detergent Fibre) and ADL (Acid Detergent Lignin) obtained on the NDF residue[3]. Starch was analysed by an enzymatic method (AFNOR, 1997) [4].

UFL = net energy for lactation (unité fourragère lait); PDI= truly digestible (dietary + microbial) protein (protéine digestible dans l'intestin)[5].

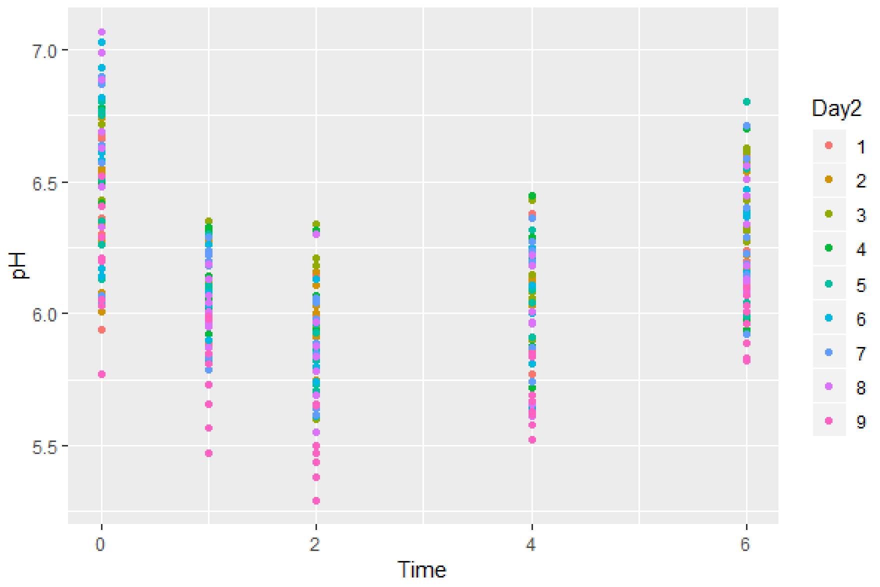

Figure S1. The post-prandial kinetics of pH before feed delivery or 1, 2, 4 and 6 hours after feed delivery. Different colours represent the nine days of sampling during the five weeks of experimentation.

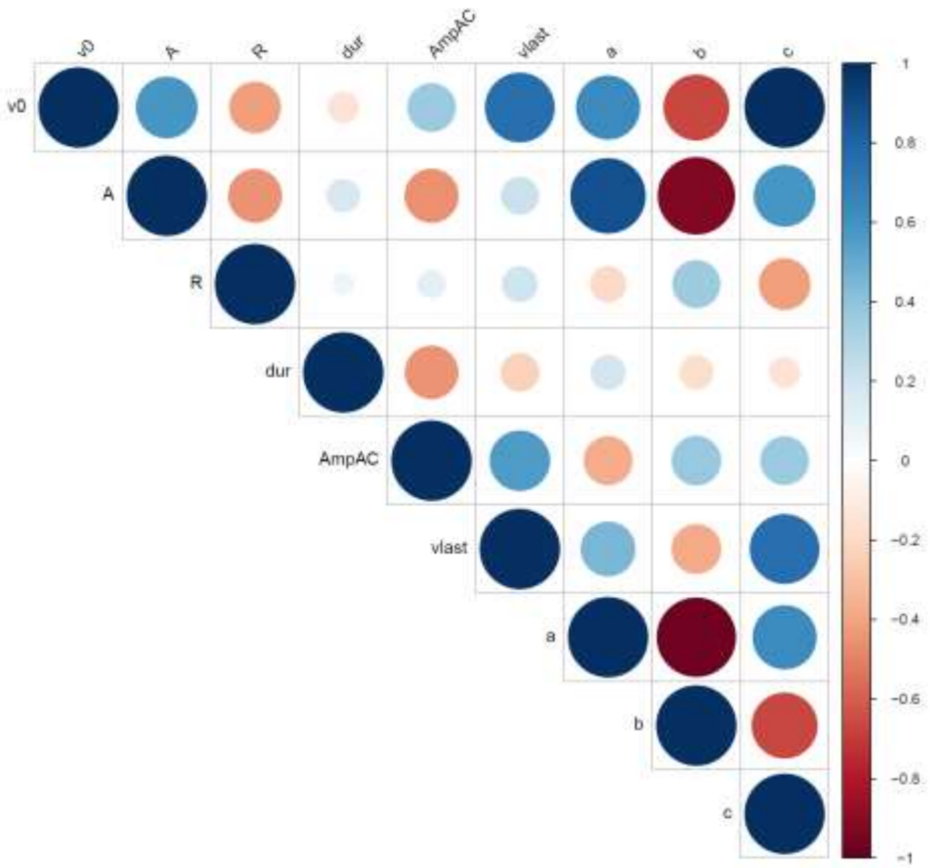

Figure S2. Correlation analysis for synthetic variables calculated for pH.

Supplementary equation 1. Reparametrization of Model 1.1

Three new and biologically meaningful parameters (v_0, A, R) were introduced to replace parametrs a, b and c of Model 1.1

$H^{+}(t) = at^2 + bt + c$, Model (1.1).

$A = a({t_{max}}^2 - t_0^2) + b(t_{max} - t_0)$

$v_0 = at_0^2 + bt_0 + c$

$R = a({t_{last}}^2 - t_0^2)/b(t_{last} - t_0)$

Model 1.2 is then resulted from integrating new definition of a, b and c in Model 1.1.

References

1. Sweeney, R. A. & Rexroad, P. R. Comparison of Leco-FP-228 nitrogen determinator with AOAC copper catalyst Kjeldahl method for crude protein. J. Assoc. Off. Anal. Chem. 70, 1028-1030, (1987).

2. Van Soest, P. J. & Wine, R. H. Use of detergents in the analysis of fibrous feeds. IV. Determination of plant cell-wall constituents. J. Assoc. Off. Anal. Chem. 50, 50-55, (1967).

3. Giger, S., Thivend, P., Sauvant, D., Dorléans, M. & Journaix, P. Etude de l'influence préalable de différents traitements amylolytiques sur la teneur en résidu NDF d'aliments du bétail. (Effect of different amylolytic pretreatments on NDF content in feedstuffs). Ann. Zootech. 36, 39-48, https://doi.org/10.1051/animres:19870104 (1987).

4. AFNOR. Aliments des animaux. Dosage de l'amidon. Méthode enzymatique. (Animal Feedingstuffs. Determination of starch content by an enzymatic method).France Patent V18-121. Paris-la Défense, France patent (1997).

5. INRA. INRA feeding system for ruminants. (Wageningen Academic Publishers, Wageningen, The Netherlands, 2018).

Corresponding author: Masoomeh.Taghipoor@inrae.fr
